# Poly[[(μ-aqua)[μ_4_-4-(carboxyl­atometh­yl)ben­zo­ato]cobalt(II)] hemi[1,4-bis­(pyridin-4-ylmeth­yl)piper­azine] hemihydrate]

**DOI:** 10.1107/S241431462300648X

**Published:** 2023-07-28

**Authors:** Gabrielle J. Gaskin, Robert L. LaDuca

**Affiliations:** a E-35 Holmes Hall, Michigan State University, Lyman Briggs College, 919 E. Shaw Lane, East Lansing, MI 48825, USA; Goethe-Universität Frankfurt, Germany

**Keywords:** crystal structure, coordination polymer, piperazine, cobalt

## Abstract

In the title com­pound, {[Co(C_9_H_6_O_4_)(H_2_O)]·0.5C_16_H_20_N_4_·0.5H_2_O}_*n*_, two-di­men­sional coordination polymer slabs are held together in the three-dimensional crystal structure by means of O—H⋯N hydrogen bonding between bound water mol­ecules and pyridyl N atoms of cocrystallized 1,4-bis­(pyridin-4-yl­meth­yl)piperazine mol­ecules.

## Abstract 2

A divalent cobalt two-dimensional slab coordination polymer with cocrystallized species, {[Co(cmb)(H_2_O)].0.5(bpmp)·0.5H_2_O}_*n*_ (where cmb is 4-(carboxyl­atometh­yl)benzoate and bpmp is 1,4-bis­(pyridin-4-ylmeth­yl)piperazine, was structurally characterized by single-crystal X-ray diffraction.

## Graphical abstract

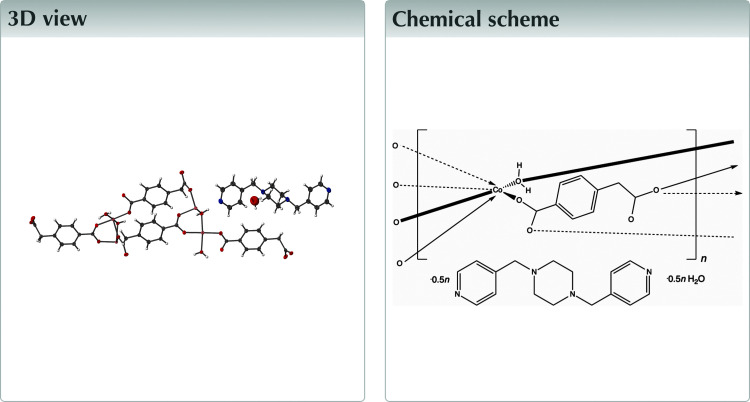

## Structure description

Our group has demonstrated the utility of 1,4-bis­(pyridin-4-ylmeth­yl)piperazine (bpmp) for the construction of divalent metal coordination polymers with a striking variety of inter­esting topologies (Robinson *et al.*, 2015 ▸). For instance, the cobalt oxalate (ox) bpmp phase {[Co(H_2_O)_4_(bpmp)](ox)}_*n*_ displays cationic one-dimensional chain motifs with unligated ox moieties. A higher temperature polymorph, {[Co(ox)(bpmp)]·3H_2_O}_*n*_, manifests a threefold inter­penetrated three-dimensional **dia** topology. Use of isophthalate (iph) as the di­carboxyl­ate ligand afforded {[Co(iph)(bpmp)]·H_2_O}_*n*_, which exhibits a dimer-based two-dimensional layered structure (Martin *et al.*, 2007 ▸). The title com­pound was isolated during an attempt to prepare a divalent cobalt coordination polymer containing both bpmp and 4-(carboxyl­atometh­yl)benzoate (cmb) ligands.

**Figure 1:**
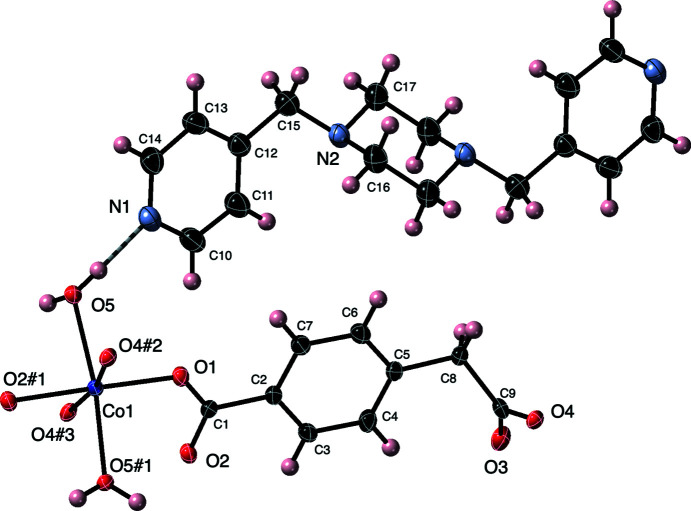
The cobalt coordination environment in the title com­pound with a full cmb ligand and cocrystallized species. Displacement ellipsoids are drawn at the 50% probability level. Water mol­ecule O1*W* has been omitted. Color code: Co dark blue, O red, N light blue, C black, and H pink. The symmetry codes are as listed in Table 1 ▸.

The asymmetric unit of the title com­pound contains a divalent Co atom, a fully deprotonated cmb ligand, a bound water mol­ecule, a cocrystallized water mol­ecule best refined at half-occupancy, and half of an unligated bpmp mol­ecule whose central piperazine ring is sited on a crystallographic inversion center. The Co atom is coordinated in a {CoO_6_} distorted octa­hedral fashion (Fig. 1 ▸) with carboxyl­ate O-atom donors from four cmb ligands in the nominal equatorial plane. The two nominally axial positions are taken up by bound water mol­ecules. Pertinent bond distances and angles for the coordination sphere are listed in Table 1 ▸.

**Figure 2:**
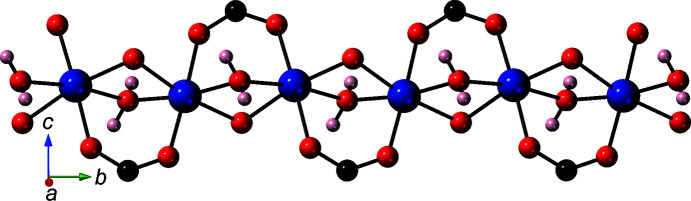
The [Co(OCO)(μ-H_2_O)(μ-O)]_*n*_ coordination polymer chain in the title com­pound.

**Figure 3:**
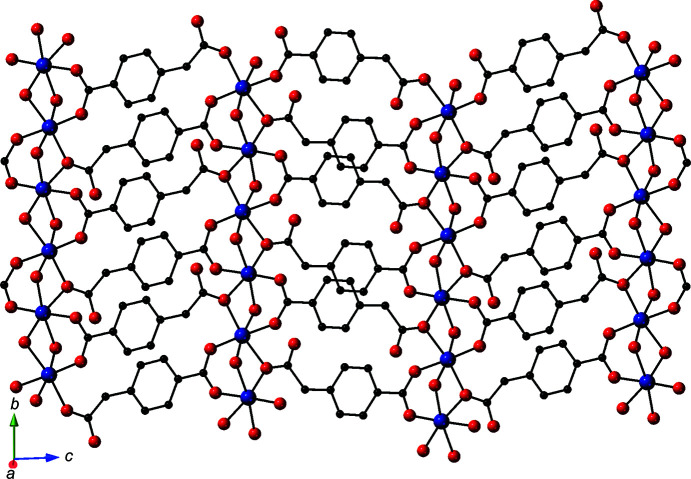
A [Co(μ-H_2_O)(cmb)]_*n*_ coordination polymer slab in the title com­pound.

The bound water mol­ecules bridge adjacent Co atoms to construct [Co(μ-H_2_O)]_*n*_ one-dimensional chain submotifs arranged parallel to the *b* crystal axis, in which the Co⋯Co inter­nuclear distance measures 3.170 (1) Å. Carboxyl­ate groups from cmb ligands and single carboxyl­ate O atoms from other cmb ligands also bridge the same Co⋯Co inter­nuclear distance, thereby affording [Co(OCO)(μ-H_2_O)(μ-O)]_*n*_ chain motifs oriented along the *b* crystal direction (Fig. 2 ▸). One carboxyl­ate terminus of the cmb ligands bridges two Co atoms in a chelating *syn*–*syn* fashion, while the other carboxyl­ate group donates a single O atom to two adjacent Co atoms. One carboxyl­ate O atom (O3) of each cmb ligand remains unligated. The chain motifs are connected into [Co_2_(μ-H_2_O)_2_(cmb)]_*n*_ two-dimensional slabs by the exo-tetra­dentate cmb ligands; these slabs are oriented parallel to the *bc* crystal planes (Fig. 3 ▸).

**Figure 4:**
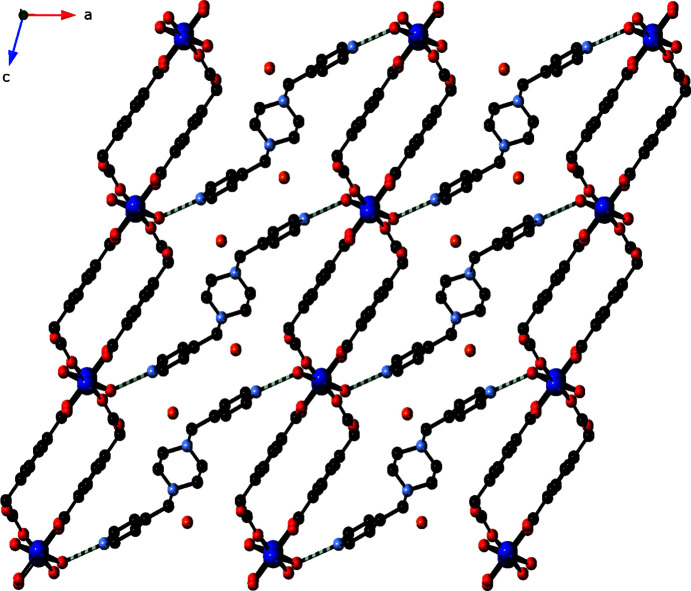
The supra­molecular three-dimensional structure formed by O—H⋯N hydrogen bonding (hatched bonds) between [Co(cmb)(μ-H_2_O)]_*n*_ slabs and cocrystallized bpmp mol­ecules.

[Co(μ-H_2_O)(cmb)]_*n*_ layers are connected into the three-dimensional crystal structure by hydrogen bonding mediated by the unligated bpmp mol­ecules in the inter­lamellar regions. The pyridyl termini of the cocrystallized bpmp mol­ecules accept hydrogen bonds from the bridging water mol­ecules in two adjacent layer motifs (Fig. 4 ▸). Cocrystallized water mol­ecules are held in the crystal by donating hydrogen bonds to the bpmp piperazine N atoms. Details regarding the hydrogen-bonding patterns in the title com­pound are listed in Table 2 ▸.

## Synthesis and crystallization

Co(NO_3_)_2_·6H_2_O (108 mg, 0.37 mmol), 4-(carb­oxy­meth­yl)benzoic acid (cmbH_2_) (67 mg, 0.37 mmol), 1,4-bis­(pyridin-4-ylmeth­yl)piperazine (bpmp) (99 mg, 0.37 mmol), and 0.75 ml of a 1.0 *M* NaOH solution were placed in 10 ml distilled H_2_O in a Teflon-lined acid digestion bomb. The bomb was sealed and heated in an oven at 393 K for 48 h and then cooled slowly to 273 K. Pink crystals of the title com­plex were obtained in 68% yield.

## Refinement

Crystal data, data collection and structure refinement details are summarized in Table 3 ▸. All H atoms attached to C atoms were placed in calculated positions and refined with a riding model. The H atoms bound to the water O5 atom were found *via* a difference map and were refined freely with a restraint of 0.85 Å for the O—H distances, but those bound to atom O1*W* were positioned geometrically and refined using a riding model.

## Supplementary Material

Crystal structure: contains datablock(s) I, global. DOI: 10.1107/S241431462300648X/bt4140sup1.cif

Structure factors: contains datablock(s) I. DOI: 10.1107/S241431462300648X/bt4140Isup2.hkl

CCDC reference: 1946425

Additional supporting information:  crystallographic information; 3D view; checkCIF report

## Figures and Tables

**Table 1:** Selected geometric parameters (Å, °)

Co1—O1	2.011 (2)	Co1—O4^iii^	2.140 (2)
Co1—O2^i^	2.029 (2)	Co1—O5	2.139 (2)
Co1—O4^ii^	2.094 (2)	Co1—O5^i^	2.169 (2)
			
O1—Co1—O2^i^	177.55 (9)	O2^i^—Co1—O5^i^	87.29 (9)
O1—Co1—O4^iii^	92.37 (9)	O4^ii^—Co1—O4^iii^	170.65 (6)
O1—Co1—O4^ii^	95.23 (9)	O4^iii^—Co1—O5^i^	93.25 (8)
O1—Co1—O5^i^	93.31 (9)	O4^ii^—Co1—O5^i^	80.90 (9)
O1—Co1—O5	92.67 (9)	O4^ii^—Co1—O5	104.48 (9)
O2^i^—Co1—O4^iii^	85.22 (9)	O5—Co1—O4^iii^	80.55 (9)
O2^i^—Co1—O4^ii^	87.21 (9)	O5—Co1—O5^i^	171.56 (4)
O2^i^—Co1—O5	86.49 (9)		

Symmetry codes: (i) 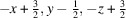 ; (ii) 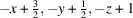 ; (iii) 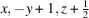 .

**Table 2:** Hydrogen-bond geometry (Å, °)

*D*—H⋯*A*	*D*—H	H⋯*A*	*D*⋯*A*	*D*—H⋯*A*
C11—H11⋯N2	0.95	2.53	2.860 (5)	101
O1*W*—H1*WA*⋯N2	0.87	2.16	2.847 (10)	136
O5—H5*A*⋯O3^i^	0.85 (2)	1.79 (2)	2.619 (3)	164 (4)
O5—H5*B*⋯N1	0.86 (2)	1.82 (2)	2.672 (4)	175 (5)

Symmetry code: (i) 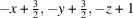 .

**Table 3:** Experimental details

Crystal data	
Chemical formula	[Co(C_9_H_6_O_4_)(H_2_O)]·0.5C_16_H_20_N_4_·0.5H_2_O
*M*_r_	796.54
Crystal system, space group	Monoclinic, *C*2/*c*
Temperature (K)	173
*a*, *b*, *c* (Å)	27.036 (6), 6.3093 (13), 20.653 (5)
β (°)	105.536 (6)
*V* (Å^3^)	3394.2 (13)
*Z*	4
Radiation type	Mo *K*α
μ (mm^−1^)	1.05
Crystal size (mm)	0.27 × 0.14 × 0.09

Data collection	
Diffractometer	Bruker APEXII CCD
Absorption correction	Multi-scan (*SADABS*; Bruker, 2014 ▸)
*T*_min_, *T*_max_	0.669, 0.745
No. of measured, independent and observed [*I* > 2σ(*I*)] reflections	8691, 3089, 2334
*R*_int_	0.044
(sin θ/λ)_max_ (Å^−1^)	0.604

Refinement	
*R*[*F* ^2^ > 2σ(*F* ^2^)], *wR*(*F* ^2^), *S*	0.041, 0.113, 1.06
No. of reflections	3089
No. of parameters	243
No. of restraints	3
H-atom treatment	H atoms treated by a mixture of independent and constrained refinement
Δρ_max_, Δρ_min_ (e Å^−3^)	0.65, −0.66

Computer programs: *COSMO* (Bruker, 2009 ▸), *SAINT* (Bruker, 2014 ▸), *SHELXT* (Sheldrick, 2015 ▸), *SHELXL* (Sheldrick, 2008 ▸), and *OLEX2* (Dolomanov *et al.*, 2009 ▸).

## full crystallographic data

### Crystal data

**Table:**

[Co(C_9_H_6_O_4_)(H_2_O)]·0.5C_16_H_20_N_4_·0.5H_2_O	*F*(000) = 1648
*M**_r_* = 796.54	*D*_x_ = 1.559 Mg m^−^^3^
Monoclinic, *C*2/*c*	Mo *K*α radiation, λ = 0.71073 Å
*a* = 27.036 (6) Å	Cell parameters from 3416 reflections
*b* = 6.3093 (13) Å	θ = 2.1–25.4°
*c* = 20.653 (5) Å	µ = 1.05 mm^−^^1^
β = 105.536 (6)°	*T* = 173 K
*V* = 3394.2 (13) Å^3^	Block, pink
*Z* = 4	0.27 × 0.14 × 0.09 mm

### Data collection

**Table:**

Bruker APEXII CCD diffractometer	3089 independent reflections
Radiation source: sealed tube	2334 reflections with *I* > 2σ(*I*)
Graphite monochromator	*R*_int_ = 0.044
Detector resolution: 8.36 pixels mm^-1^	θ_max_ = 25.4°, θ_min_ = 2.1°
ω scans	*h* = −31→32
Absorption correction: multi-scan (SADABS; Bruker, 2014)	*k* = −7→7
*T*_min_ = 0.669, *T*_max_ = 0.745	*l* = −24→24
8691 measured reflections	

### Refinement

**Table:**

Refinement on *F*^2^	Primary atom site location: dual
Least-squares matrix: full	Hydrogen site location: mixed
*R*[*F*^2^ > 2σ(*F*^2^)] = 0.041	H atoms treated by a mixture of independent and constrained refinement
*wR*(*F*^2^) = 0.113	*w* = 1/[σ^2^(*F*_o_^2^) + (0.0475*P*)^2^ + 5.8489*P*] where *P* = (*F*_o_^2^ + 2*F*_c_^2^)/3
*S* = 1.06	(Δ/σ)_max_ = 0.002
3089 reflections	Δρ_max_ = 0.65 e Å^−^^3^
243 parameters	Δρ_min_ = −0.66 e Å^−^^3^
3 restraints	

### Special details

**Table:**

Experimental. Data was collected using a BRUKER CCD (charge coupled device) based diffractometer equipped with an Oxford low-temperature apparatus operating at 173 K. A suitable crystal was chosen and mounted on a nylon loop using Paratone oil. Data were measured using omega scans of 0.5° per frame for 30 s. The total number of images were based on results from the program COSMO where redundancy was expected to be 4 and completeness to 0.83Å to 100%. Cell parameters were retrieved using APEX II software and refined using SAINT on all observed reflections.Data reduction was performed using the SAINT software which corrects for Lp. Scaling and absorption corrections were applied using SADABS6 multi-scan technique, supplied by George Sheldrick. The structure was solved by the direct method using the SHELXT program and refined by least squares method on F2, SHELXL, incorporated in OLEX2.
Geometry. All esds (except the esd in the dihedral angle between two l.s. planes) are estimated using the full covariance matrix. The cell esds are taken into account individually in the estimation of esds in distances, angles and torsion angles; correlations between esds in cell parameters are only used when they are defined by crystal symmetry. An approximate (isotropic) treatment of cell esds is used for estimating esds involving l.s. planes.
Refinement. The structure was refined by Least Squares using version 2018/3 of XL (Sheldrick, 2015) incorporated in Olex2 (Dolomanov *et al.*, 2009). All non-hydrogen atoms were refined anisotropically. Hydrogen atom positions were calculated geometrically and refined using the riding model, except for the Hydrogen atom on the nitrogen atom which was found by difference Fourier methods and refined isotropically.

### Fractional atomic coordinates and isotropic or equivalent isotropic displacement parameters (Å^2^)

**Table:**

	*x*	*y*	*z*	*U*_iso_*/*U*_eq_	Occ. (<1)
Co1	0.74834 (2)	0.12245 (6)	0.74217 (2)	0.01613 (16)	
O1	0.77518 (9)	0.2010 (3)	0.66350 (10)	0.0218 (5)	
O2	0.77731 (9)	0.5545 (3)	0.67641 (10)	0.0214 (5)	
O3	0.84413 (9)	0.8638 (3)	0.37647 (11)	0.0251 (6)	
O4	0.79387 (8)	0.6398 (3)	0.30472 (10)	0.0177 (5)	
O5	0.69426 (9)	0.3776 (3)	0.72357 (11)	0.0176 (5)	
N1	0.61639 (12)	0.3261 (5)	0.77903 (15)	0.0320 (7)	
N2	0.50168 (11)	0.4195 (5)	0.93570 (14)	0.0300 (7)	
C1	0.78312 (12)	0.3868 (5)	0.64604 (14)	0.0174 (7)	
C2	0.80076 (12)	0.4150 (5)	0.58368 (15)	0.0200 (7)	
C3	0.80611 (13)	0.6162 (5)	0.56001 (16)	0.0222 (7)	
H3	0.799069	0.736233	0.583858	0.027*	
C4	0.82169 (14)	0.6449 (5)	0.50162 (16)	0.0272 (8)	
H4	0.824641	0.784229	0.485601	0.033*	
C5	0.83291 (12)	0.4727 (5)	0.46672 (14)	0.0185 (7)	
C6	0.82768 (13)	0.2713 (5)	0.49056 (16)	0.0255 (8)	
H6	0.835191	0.151654	0.466903	0.031*	
C7	0.81175 (13)	0.2408 (5)	0.54812 (16)	0.0251 (8)	
H7	0.808268	0.101157	0.563545	0.030*	
C8	0.85144 (13)	0.4970 (5)	0.40366 (14)	0.0202 (7)	
H8A	0.889229	0.513829	0.417283	0.024*	
H8B	0.843514	0.364930	0.377015	0.024*	
C9	0.82837 (13)	0.6809 (5)	0.35940 (15)	0.0195 (7)	
C10	0.60190 (15)	0.4890 (7)	0.81086 (19)	0.0395 (10)	
H10	0.617359	0.623322	0.809065	0.047*	
C11	0.56540 (16)	0.4711 (6)	0.8464 (2)	0.0419 (10)	
H11	0.556036	0.591621	0.867983	0.050*	
C12	0.54265 (14)	0.2779 (6)	0.85029 (17)	0.0304 (8)	
C13	0.55802 (15)	0.1093 (6)	0.81812 (19)	0.0355 (9)	
H13	0.543538	−0.027144	0.819787	0.043*	
C14	0.59459 (15)	0.1393 (6)	0.78341 (19)	0.0354 (9)	
H14	0.604698	0.020841	0.761580	0.043*	
C15	0.50169 (15)	0.2492 (7)	0.88805 (19)	0.0373 (10)	
H15A	0.467525	0.242547	0.855163	0.045*	
H15B	0.507536	0.112621	0.912525	0.045*	
C16	0.54563 (14)	0.4017 (6)	0.99510 (18)	0.0345 (9)	
H16A	0.577929	0.403010	0.981173	0.041*	
H16B	0.543748	0.265931	1.018344	0.041*	
C17	0.45449 (14)	0.4153 (7)	0.95746 (19)	0.0371 (10)	
H17A	0.452101	0.279572	0.980461	0.045*	
H17B	0.424369	0.426346	0.917854	0.045*	
O1W	0.4535 (4)	0.7370 (16)	0.8420 (5)	0.131 (4)	0.5
H1WA	0.455906	0.672793	0.879981	0.196*	0.5
H1WB	0.468736	0.858073	0.853331	0.196*	0.5
H5A	0.6813 (14)	0.440 (6)	0.6864 (13)	0.044 (12)*	
H5B	0.6705 (14)	0.359 (7)	0.743 (2)	0.072 (17)*	

### Atomic displacement parameters (Å^2^)

**Table:**

	*U*^11^	*U*^22^	*U*^33^	*U*^12^	*U*^13^	*U*^23^
Co1	0.0225 (3)	0.0114 (2)	0.0161 (2)	−0.00011 (18)	0.00797 (18)	0.00042 (16)
O1	0.0344 (15)	0.0149 (12)	0.0194 (11)	0.0002 (10)	0.0130 (10)	0.0009 (9)
O2	0.0313 (14)	0.0169 (12)	0.0196 (11)	−0.0004 (10)	0.0132 (10)	0.0000 (9)
O3	0.0346 (15)	0.0200 (13)	0.0178 (11)	−0.0065 (11)	0.0019 (10)	0.0005 (9)
O4	0.0228 (13)	0.0150 (12)	0.0127 (10)	−0.0003 (9)	0.0003 (9)	−0.0008 (8)
O5	0.0221 (13)	0.0143 (12)	0.0168 (11)	0.0021 (10)	0.0061 (10)	0.0019 (9)
N1	0.0281 (19)	0.0404 (19)	0.0293 (16)	0.0012 (15)	0.0109 (14)	0.0035 (14)
N2	0.0225 (17)	0.0423 (19)	0.0271 (15)	−0.0019 (14)	0.0101 (13)	−0.0023 (13)
C1	0.0181 (18)	0.0184 (17)	0.0152 (15)	0.0010 (14)	0.0037 (13)	0.0017 (13)
C2	0.0214 (19)	0.0233 (18)	0.0160 (15)	0.0024 (14)	0.0059 (13)	0.0013 (13)
C3	0.034 (2)	0.0137 (16)	0.0223 (16)	0.0006 (15)	0.0127 (15)	−0.0021 (13)
C4	0.045 (2)	0.0169 (18)	0.0232 (17)	−0.0018 (16)	0.0160 (16)	0.0033 (14)
C5	0.0194 (18)	0.0206 (17)	0.0148 (14)	−0.0006 (14)	0.0034 (13)	−0.0001 (13)
C6	0.036 (2)	0.0184 (18)	0.0257 (17)	0.0017 (16)	0.0138 (16)	−0.0015 (14)
C7	0.037 (2)	0.0154 (17)	0.0270 (17)	0.0014 (16)	0.0160 (16)	0.0031 (14)
C8	0.0246 (19)	0.0197 (17)	0.0171 (15)	0.0014 (15)	0.0069 (14)	0.0028 (13)
C9	0.0214 (19)	0.0230 (18)	0.0172 (15)	−0.0015 (14)	0.0104 (14)	−0.0021 (13)
C10	0.046 (3)	0.035 (2)	0.043 (2)	−0.004 (2)	0.022 (2)	0.0025 (19)
C11	0.053 (3)	0.034 (2)	0.050 (2)	−0.002 (2)	0.032 (2)	−0.006 (2)
C12	0.028 (2)	0.035 (2)	0.0288 (18)	−0.0014 (18)	0.0088 (16)	0.0007 (16)
C13	0.038 (2)	0.030 (2)	0.042 (2)	−0.0035 (18)	0.0172 (18)	0.0008 (17)
C14	0.040 (2)	0.034 (2)	0.036 (2)	0.0052 (19)	0.0168 (18)	−0.0008 (17)
C15	0.036 (2)	0.043 (2)	0.037 (2)	−0.005 (2)	0.0162 (18)	−0.0036 (18)
C16	0.022 (2)	0.050 (3)	0.033 (2)	0.0060 (18)	0.0099 (16)	−0.0004 (18)
C17	0.020 (2)	0.061 (3)	0.0313 (19)	−0.0041 (19)	0.0076 (16)	−0.0082 (18)
O1W	0.165 (10)	0.120 (8)	0.115 (8)	−0.008 (8)	0.052 (7)	0.013 (7)

### Geometric parameters (Å, º)

**Table:**

Co1—O1	2.011 (2)	C5—C8	1.523 (4)
Co1—O2^i^	2.029 (2)	C6—H6	0.9500
Co1—O4^ii^	2.094 (2)	C6—C7	1.382 (4)
Co1—O4^iii^	2.140 (2)	C7—H7	0.9500
Co1—O5	2.139 (2)	C8—H8A	0.9900
Co1—O5^i^	2.169 (2)	C8—H8B	0.9900
O1—C1	1.262 (4)	C8—C9	1.505 (4)
O2—C1	1.261 (4)	C10—H10	0.9500
O3—C9	1.248 (4)	C10—C11	1.383 (5)
O4—C9	1.284 (4)	C11—H11	0.9500
O5—H5A	0.851 (19)	C11—C12	1.377 (5)
O5—H5B	0.855 (19)	C12—C13	1.376 (5)
N1—C10	1.334 (5)	C12—C15	1.526 (5)
N1—C14	1.332 (5)	C13—H13	0.9500
N2—C15	1.457 (5)	C13—C14	1.381 (5)
N2—C16	1.466 (5)	C14—H14	0.9500
N2—C17	1.462 (4)	C15—H15A	0.9900
C1—C2	1.499 (4)	C15—H15B	0.9900
C2—C3	1.382 (4)	C16—H16A	0.9900
C2—C7	1.397 (4)	C16—H16B	0.9900
C3—H3	0.9500	C16—C17^iv^	1.515 (5)
C3—C4	1.392 (4)	C17—H17A	0.9900
C4—H4	0.9500	C17—H17B	0.9900
C4—C5	1.382 (4)	O1W—H1WA	0.8698
C5—C6	1.384 (5)	O1W—H1WB	0.8699
			
O1—Co1—O2^i^	177.55 (9)	C7—C6—H6	119.4
O1—Co1—O4^iii^	92.37 (9)	C2—C7—H7	119.9
O1—Co1—O4^ii^	95.23 (9)	C6—C7—C2	120.1 (3)
O1—Co1—O5^i^	93.31 (9)	C6—C7—H7	119.9
O1—Co1—O5	92.67 (9)	C5—C8—H8A	108.6
O2^i^—Co1—O4^iii^	85.22 (9)	C5—C8—H8B	108.6
O2^i^—Co1—O4^ii^	87.21 (9)	H8A—C8—H8B	107.5
O2^i^—Co1—O5	86.49 (9)	C9—C8—C5	114.8 (3)
O2^i^—Co1—O5^i^	87.29 (9)	C9—C8—H8A	108.6
O4^ii^—Co1—O4^iii^	170.65 (6)	C9—C8—H8B	108.6
O4^iii^—Co1—O5^i^	93.25 (8)	O3—C9—O4	123.2 (3)
O4^ii^—Co1—O5^i^	80.90 (9)	O3—C9—C8	119.1 (3)
O4^ii^—Co1—O5	104.48 (9)	O4—C9—C8	117.7 (3)
O5—Co1—O4^iii^	80.55 (9)	N1—C10—H10	118.5
O5—Co1—O5^i^	171.56 (4)	N1—C10—C11	122.9 (4)
C1—O1—Co1	125.86 (19)	C11—C10—H10	118.5
C1—O2—Co1^v^	134.8 (2)	C10—C11—H11	120.1
Co1^ii^—O4—Co1^vi^	96.93 (8)	C12—C11—C10	119.8 (4)
C9—O4—Co1^ii^	138.0 (2)	C12—C11—H11	120.1
C9—O4—Co1^vi^	123.7 (2)	C11—C12—C15	122.1 (3)
Co1—O5—Co1^v^	94.73 (9)	C13—C12—C11	117.3 (3)
Co1—O5—H5A	127 (3)	C13—C12—C15	120.6 (3)
Co1^v^—O5—H5A	93 (3)	C12—C13—H13	120.2
Co1^v^—O5—H5B	120 (3)	C12—C13—C14	119.7 (4)
Co1—O5—H5B	112 (3)	C14—C13—H13	120.2
H5A—O5—H5B	108 (4)	N1—C14—C13	123.2 (4)
C14—N1—C10	117.0 (3)	N1—C14—H14	118.4
C15—N2—C16	111.2 (3)	C13—C14—H14	118.4
C15—N2—C17	110.6 (3)	N2—C15—C12	112.9 (3)
C17—N2—C16	108.7 (3)	N2—C15—H15A	109.0
O1—C1—C2	118.3 (3)	N2—C15—H15B	109.0
O2—C1—O1	125.7 (3)	C12—C15—H15A	109.0
O2—C1—C2	116.0 (3)	C12—C15—H15B	109.0
C3—C2—C1	120.0 (3)	H15A—C15—H15B	107.8
C3—C2—C7	118.7 (3)	N2—C16—H16A	109.7
C7—C2—C1	121.3 (3)	N2—C16—H16B	109.7
C2—C3—H3	119.6	N2—C16—C17^iv^	110.0 (3)
C2—C3—C4	120.7 (3)	H16A—C16—H16B	108.2
C4—C3—H3	119.6	C17^iv^—C16—H16A	109.7
C3—C4—H4	119.7	C17^iv^—C16—H16B	109.7
C5—C4—C3	120.6 (3)	N2—C17—C16^iv^	109.8 (3)
C5—C4—H4	119.7	N2—C17—H17A	109.7
C4—C5—C6	118.6 (3)	N2—C17—H17B	109.7
C4—C5—C8	122.4 (3)	C16^iv^—C17—H17A	109.7
C6—C5—C8	119.0 (3)	C16^iv^—C17—H17B	109.7
C5—C6—H6	119.4	H17A—C17—H17B	108.2
C7—C6—C5	121.3 (3)	H1WA—O1W—H1WB	104.5
			
Co1—O1—C1—O2	2.7 (5)	C5—C6—C7—C2	−0.3 (5)
Co1—O1—C1—C2	−176.9 (2)	C5—C8—C9—O3	76.4 (4)
Co1^v^—O2—C1—O1	−3.5 (5)	C5—C8—C9—O4	−105.1 (3)
Co1^v^—O2—C1—C2	176.2 (2)	C6—C5—C8—C9	145.6 (3)
Co1^vi^—O4—C9—O3	−11.0 (4)	C7—C2—C3—C4	0.6 (5)
Co1^ii^—O4—C9—O3	−174.2 (2)	C8—C5—C6—C7	179.0 (3)
Co1^vi^—O4—C9—C8	170.56 (19)	C10—N1—C14—C13	0.8 (6)
Co1^ii^—O4—C9—C8	7.3 (4)	C10—C11—C12—C13	0.3 (6)
O1—C1—C2—C3	175.1 (3)	C10—C11—C12—C15	−179.3 (4)
O1—C1—C2—C7	−4.5 (5)	C11—C12—C13—C14	−0.5 (6)
O2—C1—C2—C3	−4.6 (5)	C11—C12—C15—N2	−18.8 (5)
O2—C1—C2—C7	175.8 (3)	C12—C13—C14—N1	−0.1 (6)
N1—C10—C11—C12	0.5 (6)	C13—C12—C15—N2	161.7 (3)
C1—C2—C3—C4	−179.0 (3)	C14—N1—C10—C11	−1.0 (6)
C1—C2—C7—C6	179.6 (3)	C15—N2—C16—C17^iv^	178.2 (3)
C2—C3—C4—C5	−1.0 (5)	C15—N2—C17—C16^iv^	−177.9 (3)
C3—C2—C7—C6	0.1 (5)	C15—C12—C13—C14	179.1 (4)
C3—C4—C5—C6	0.8 (5)	C16—N2—C15—C12	−73.7 (4)
C3—C4—C5—C8	−178.3 (3)	C16—N2—C17—C16^iv^	59.7 (4)
C4—C5—C6—C7	−0.2 (5)	C17—N2—C15—C12	165.4 (3)
C4—C5—C8—C9	−35.2 (5)	C17—N2—C16—C17^iv^	−59.8 (4)

Symmetry codes: (i) −*x*+3/2, *y*−1/2, −*z*+3/2; (ii) −*x*+3/2, −*y*+1/2, −*z*+1; (iii) *x*, −*y*+1, *z*+1/2; (iv) −*x*+1, −*y*+1, −*z*+2; (v) −*x*+3/2, *y*+1/2, −*z*+3/2; (vi) *x*, −*y*+1, *z*−1/2.

### Hydrogen-bond geometry (Å, º)

**Table:**

*D*—H···*A*	*D*—H	H···*A*	*D*···*A*	*D*—H···*A*
C11—H11···N2	0.95	2.53	2.860 (5)	101
O1*W*—H1*WA*···N2	0.87	2.16	2.847 (10)	136
O5—H5*A*···O3^vii^	0.85 (2)	1.79 (2)	2.619 (3)	164 (4)
O5—H5*B*···N1	0.86 (2)	1.82 (2)	2.672 (4)	175 (5)

Symmetry code: (vii) −*x*+3/2, −*y*+3/2, −*z*+1.
